# Calibrating an Ionosonde for Ionospheric Attenuation Measurements

**DOI:** 10.3390/s18051564

**Published:** 2018-05-15

**Authors:** Lorenzo Gilli, Umberto Sciacca, Enrico Zuccheretti

**Affiliations:** Istituto Nazionale di Geofisica e Vulcanologia, 00143 Rome, Italy; lorenzo.lw89@gmail.com (L.G.); enrico.zuccheretti@ingv.it (E.Z.)

**Keywords:** ionosonde, ionospheric attenuation, calibration

## Abstract

Vertical ionospheric soundings have been performed at almost all ionospheric observatories with little attention to measuring the attenuation of the signal between transmission and reception. When the absorption has been determined, this has been achieved by comparing the received power after the first and second reflections, but this method has some limitations due to the unknown reflection coefficient of the ground and the non-continuous presence of the second reflection. This paper deals with a different method based on precise calibration of the sounding system, allowing determination of absolute signal attenuation after a single reflection. This approach is affected by a systematic error due to imperfect calibration of the antennas, but when the focus of interest is to measure a trend over a specified period, it is very accurate. The article describes how calibration was implemented, the measurement output formats, and finally it presents some results from a meaningful set of measurements in order to demonstrate what this method can accomplish.

## 1. Introduction

One of the most widely used methods to study the ionosphere is vertical sounding, in particular to determine the electron density profile as a function of height. For this purpose it has always been sufficient to measure the “virtual” height at which an electromagnetic wave, transmitted perpendicular to the ground, appears to reflect. It is called “virtual” height because a propagation velocity equal to that of vacuum is assumed. Based on the knowledge of reflection heights, which vary with frequency, it is possible to obtain an electron density profile. The special type of radar used for this kind of measurement is called an ionosonde and it does not need to measure the intensity of the reflected echo, but only detect the presence of an echo and measure the delay relative to the transmitted signal [1].

However, a knowledge of the intensity of the reflected signal could be exploited to deduce the attenuation along the path between the transmitter and receiver. This attenuation is influenced mainly by the length of the path (“geometric” attenuation), which is easy to calculate and so can be eliminated from the total attenuation, with the remainder due to propagation in the ionospheric medium.

Establishing an absorption value is of interest because it can be used to test ionospheric models. Many theoretical studies have dealt with this topic in various ways and some examples from the huge literature on the subject are as follows. An attempt was made to find connections between absorption and electron collision frequency, based on the concentration of neutral particles and electrons in the ionospheric layers [2]. Other studies have calculated total absorption and its trend inside individual layers [3], identifying differences between the D, E, and F layers [4,5]. A theoretical investigation was made to establish the contribution of deviative and non-deviative components to total absorption, also assessing seasonal evolution [6]. The presence of the sporadic-E layer makes prediction more difficult because of its variable characteristics, ranging from situations in which it blankets the higher layers, to others in which it is more transparent [7]. In some advanced models the consequences of a possible splitting of the D layer have been studied [8] and this could be assisted by the availability of absorption measurements. More recently new theoretical models [9] and numerical methods have been proposed for forecasting absorption using a computer [10].

The theoretical studies have been associated with numerous investigations on actual measurements [11], especially as regards dependency on frequency and season [12,13]. Particular interest has been paid to the D, E, and sporadic-E layers [14,15]. Recently absorption measurements have been used to study disturbances [16]. Particular attention has been paid to situation in which attenuation is subject to relevant variations over short time periods, like sunrise and sunset [17] or during solar eclipses [18,19].

These numerous studies indicate the great interest in studying ionospheric absorption. Various methods have been devised to make actual measurements and the reference paper for this remains [20], which discusses many methods, in particular the one named “A1”. This is the most widely used in diverse works, including [21,22], and concentrates more than the others on the measurement methodology. This type of measurement is difficult because an accurate determination of absorption requires a comparison between the true received and transmitted power of the signals. The term “true” means an exact knowledge of the power of the electromagnetic wave that leaves (or enters) the antennas, which is useful to far field propagation. A knowledge of the power leaving the amplifier (or entering the receiver) is not useful. There are essentially two problems: an imperfect knowledge of the parameters of the radar system used for measurement, and possible variation in these parameters even over relatively short time periods (i.e., hours). Among the system parameters, the characteristics of the antennas are the most difficult to define.

In order to overcome these problems a comparative method had to be applied: the relative amplitudes of the signals received after a first and second reflection were measured and then a relatively simple elaboration of the result allowed determination of the actual absorption [21]. This method has the advantage of eliminating the problems mentioned above regarding the determination of system parameters. However, it also subject to three possible critical issues: (a) it is assumed that the reflection off the ground between the first and the second reflections off the ionosphere takes place without loss (i.e., all the incident energy is reflected back towards the ionosphere); (b) the second reflection has to actually take place and be detected, which does not always occur because often absorption is so great that the signal is no longer detectable after the first reflection; (c) in systems that use linear polarisation antennas, the polarisation plane rotates every times a signal undergoes an ionospheric reflection; this causes a gain (or loss, depending on the antenna system) which is extremely difficult to predict and after a second reflection becomes varied in an unpredictable way.

The need resolve these critical issues induce the authors to try and determine absorption using a direct method, which involves calculating or measuring all the parameters characterising an ionosonde by calibrating the receiver, transmitter, and antennas in a particular way. This was possible because all the parameters of the system being used (like all modern systems) are essentially constant, or vary so slowly that a calibration can be used for months before it has to be repeated. Obviously some residual errors persist (due to the imperfect characterisation of the antennas, as will be seen), but they affect an absolute measure of ionospheric absorption. If the interest is a comparison between attenuation values at different times (at the same frequency), the errors compensate and the measurement can be considered very accurate. This is all achieved without the constraint of requiring a second reflection.

This work does not intend to present any relevant results obtained in ionospheric absorption measurements. Its purpose is mainly technical, with the aim of describing the procedures that enable absorption measurement. After a brief description of the ionosonde used, the measuring principle and calibration procedure will be described. Next, various ways of presenting the measurement data obtained from the calibration will be presented, together with some actual measurements.

## 2. The AIS-INGV Ionosonde

**Figure 1:**
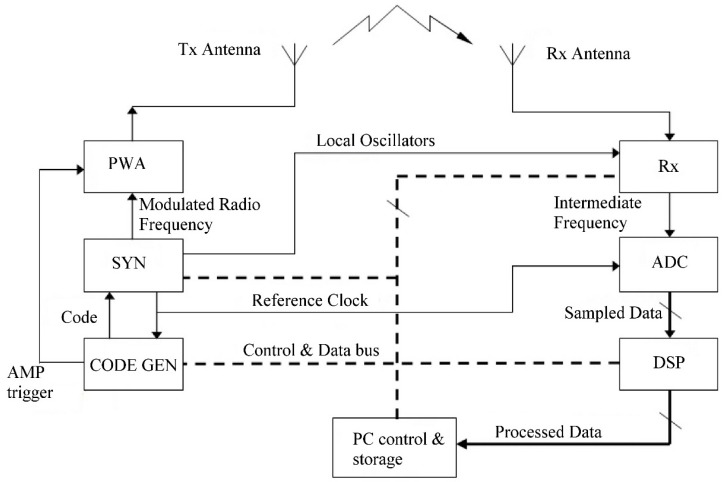
Simplified diagram of AIS-INGV ionosonde, showing the main functional subsystems: PWA is the power amplifier, Rx is the receiver, SYN is the frequency synthesiser, ADC is the Analog to Digital Converter, CODE GEN is the code generator, DSP is the Digital Signal Processor (for details on their operation see the text).

The AIS-INGV ionosonde (AIS means Advanced Ionospheric Sounder, INGV is the acronym of Istituto Nazionale di Geofisica e Vulcanologia, the Italian National Institution for Geophysics and Volcanology) usually employed for the routine vertical sounding at Rome ionospheric observatory is composed by many subsections that will be now briefly described (see Figure 1).

The ionosonde consists of two main sections, which approximately correspond to its transmitting and receiving functionality: on the left, a code generator (CODE GEN), a frequency synthesiser (SYN), and a power amplifier (PWA); on the right, a radio receiver (Rx), an analogue to digital converter (ADC), and a digital signal processor (DSP). Each block is controlled by a personal computer (PC control and storage), which can store and display data. The antennas complete the system: they are both progressive wave wide band delta antennas.

The frequency synthesiser uses three Direct Digital Synthesis (DDS) devices to generate all the reference signals in the system, based on a unique reference quartz oscillator. Three are local oscillator frequencies sent to the receiver, one is the transmission carrier, the last one is a 400 kHz reference clock used to synchronise the ADC and create the codes.

The ionosonde makes use of a pulse compression technique to increase the signal-to-noise ratio without increasing the peak transmitted power. The compression is accomplished by superimposing a bi-phase digital complementary code on the carrier of the transmitted signal. The sequence of 0’s and 1’s that constitute the code are programmed by the PC and generated by the CODE GEN block; the final mixing of the code with the carrier is performed inside the SYN block. The CODE GEN also generates a trigger to be sent to the power amplifier, which actually sends the encoded carrier to the transmitting antenna only when the trigger signal enables transmission.

After reflecting off the ionosphere the echo returns to the receiving antenna and receiver. At the receiver input a group of filters selects only a fraction of the HF band: the transmitted frequency is varied during a sounding (usually between 1 and 20 MHz), and so in order to limit the total amount of noise entering the receiver, relatively narrow band filters are selected in sequence while the scan proceeds. The receiver then amplifies the signal, together with three down conversions, which filter out the real and image interferences. In order to make the receiver work best in various operating conditions, it is provided with variable gain, selectable using the PC. It is a fixed parameter with no real-time adaptive gain. The receiver output is then split into two channels and quadrature sampling is performed. The digital data stream is finally sent to the DSP, which makes the calculations required to build an ionogram (FFT, filtering and correlation with the code). A sum of subsequent echoes (integration) is performed to increase the signal to noise ratio. This parameter (the number of echoes summed) is also fixed and selectable depending on the operating conditions.

**Figure 2:**
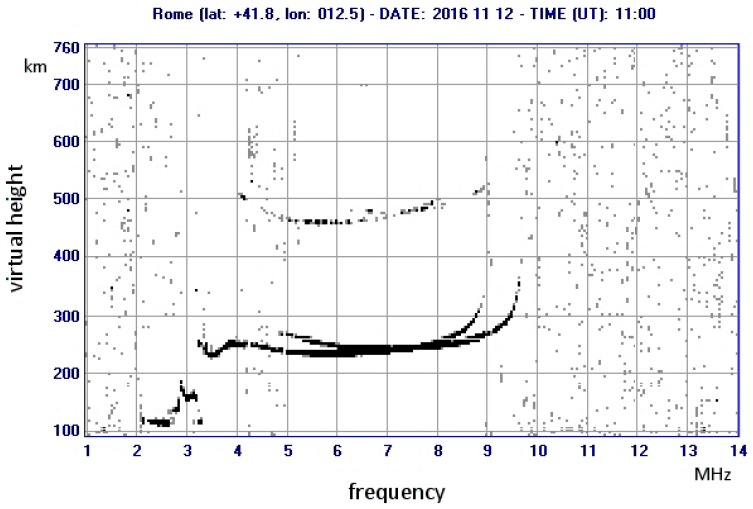
A typical ionogram, obtained by the AIS ionosonde on 12 November 2016 at Rome Observatory. The traces reveal the presence of the E-layer (2–3 MHz) and the F layers from 3.5 to 9 MHz. The presence of two traces for the F2 layer is due to the Earth magnetic field that makes the different polarisation modes propagate at two different speeds. It is visible also a weak second reflection (at about 460–500 km).

The PC uses the raw data to draw the ionogram, while also sending the appropriate programming information to all the blocks. A second version of the ionosonde has been engineered, in which the function of the DSP is included inside the PC as software routines. A more detailed description of the various boards comprising the system is reported in [23]. A typical ionogram produced by the ionosonde is shown in Figure 2.

In the graphic output, the vertical axis represents the measured heights at which reflections took place at a corresponding frequency on the horizontal axis. The heights are not real (they are called “virtual” heights) and they are estimated based on the measured delay times between transmission and reception, assuming the radio waves travel at the speed of light in vacuum. The true heights of the reflecting layers can be calculated by processing the ionogram, extracting also the actual values of electron density vs. height. To carry out this task, no information about the strength of the received signal is needed, only the information included in a standard ionogram, i.e., the position (delay) of the echo or echoes for each frequency. Nonetheless, a knowledge of the attenuation of the signal could add interesting information to the sounding. The extraction of such information is in principle possible because the system output, after being processed by the DSP, is a function of the amplitude of the received signal.

## 3. Attenuation Measurement Principles and Calibration of the AIS-INGV Ionosonde

**Figure 3:**
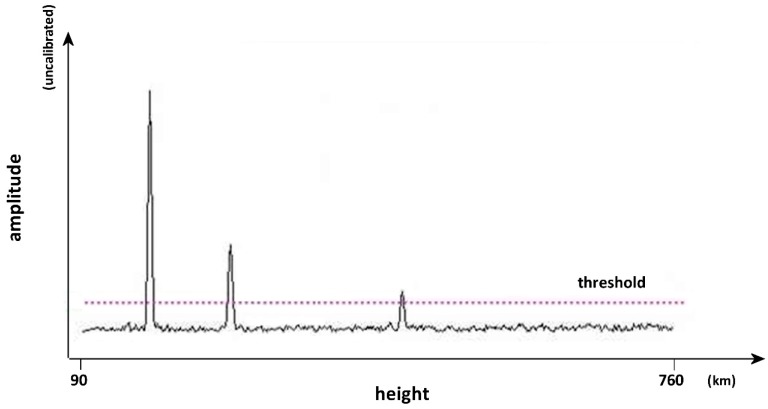
An example of a received trace at a fixed frequency. The instantaneous amplitude is compared to a threshold and only the peaks above the threshold are recognised as echoes.

The ionosonde system has always been able to measure the amplitudes of received signals, provided that the echo signal was correctly detected. Representing a single time response to a transmitted pulse at a fixed frequency produces a trace like the one shown in Figure 3. There are various algorithms that can be used to detect an echo, but basically all of them compare the instant amplitude of the received signal against a threshold: if the signal is greater an echo is recognised. The three peaks in Figure 3 represent different echoes due to multiple reflections, recognised as true echoes because the amplitude is above the threshold (of course “false positives” are possible, like the third peak, which exceeds the threshold but is not an echo). The threshold can be determined in many ways, usually an algorithm is used to calculate its value in correspondence to the actual signal-to-noise ratio and the wanted probability of detection, maintaining a low and fixed probability of false alarm. In the AIS ionosonde the level of the threshold is proportional to the noise floor level at each frequency, although the constant of proportionality is determined empirically so that acceptable traces are obtained.

**Figure 4:**
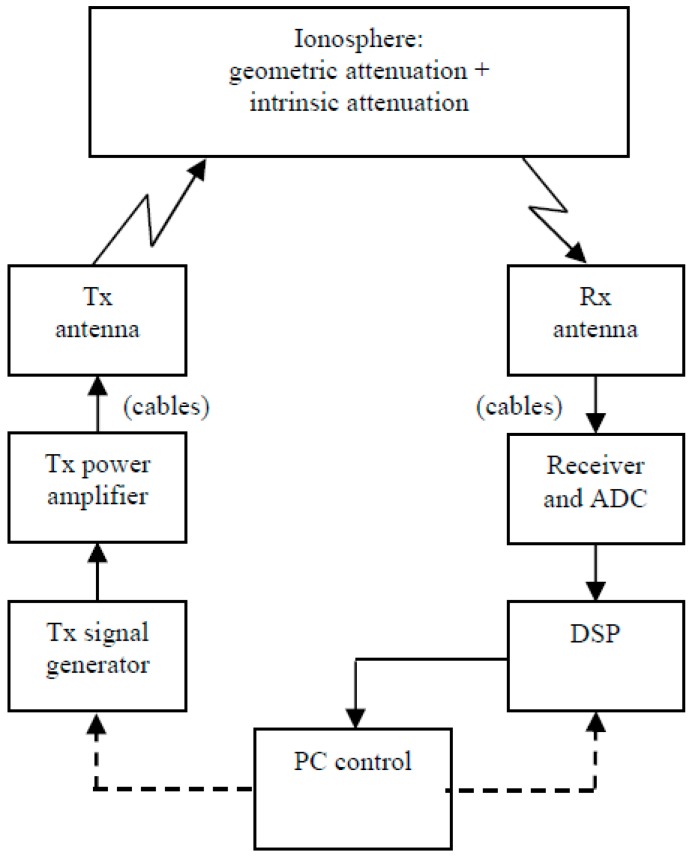
Simplified diagram of the ionosonde calibration setup. In an ideal setup the transmitting and receiving antennas should be connected directly, but this is not possible because of the intrinsic nature of the antenna-transmission medium coupling, so the antennas are excluded from the calibration setup and they have to be characterised by theoretical models.

Therefore, in principle the amplitude of the signal is already used, but it has never been scaled and the only assumption is that greater output corresponds with greater input to the receiver. In order to obtain an absolute (or even relative) measurement of received amplitude, the system needs to be calibrated. The block diagram in Figure 1 can be further simplified to highlight the essential factors that influence measurement, as shown in Figure 4.

The attenuation affecting a signal during its journey from transmitting to receiving antenna is caused by both geometric attenuation, and “intrinsic” attenuation (deviative and non-deviative). Intrinsic attenuation is the most interesting of the two, while the value of the geometric attenuation can be determined by knowing the height of the reflecting layer, which is obtained by traditional sounding. These steps regard the post processing calculations, which can be made after determining the total attenuation through accurate measurement of the received signal. Perfect calibration could be achieved by directly connecting the transmitter output to the receiver input, excluding the propagation medium.

In principle, a direct connection between the Tx and Rx antenna would allow measurement of the outputs for various signals at different power levels. Unfortunately such a connection is impossible, due to the way that antennas radiate electromagnetic energy into free space, exploiting the so called “far-field” radiation mode, while a more direct link would require a “near-field” mode. Currently, the measurement of the far field radiation pattern of the antennas used is not feasible, due to their large dimensions (some tens of metres, requiring an anechoic chamber some kilometres in size) so the only way available to take into account the contribution of antennas is to simulate their behaviour, but again in this case the results can be affected by systematic errors that are difficult to predict.

Excluding the antennas, all the remaining components of the system can be characterised by direct measurement: the Tx power amplifier, the cables, and the receiver. The latter is the most problematic to calibrate because in general its response is not linear, making an accurate evaluation of its response necessary.

The system output during a normal sounding can be summarised by the following expression:

(1) $$M(f,t,a)=\frac{P_{t}(f)}{A_{g}(f,t)\cdot A_{ion}(f,t)\cdot A_{ant}(f)\cdot A_{cable}(f)}\cdot F_{rx}(f,a)$$

where: *f* is the sounding frequency, *t* is the time, *a* is the amplitude (to be taken into account when a subsystem has not a linear behaviour), *M* is the power measured at the end of the processing procedures, *P_t_* is the transmitted power, *A_g_* is the geometric attenuation, *A_ion_* is the ionospheric attenuation, *A_ant_* is the total attenuation (or gain) of antennas, assumed to be equal, *A_cable_* is the attenuation of all cables, *F_rx_* is the transfer function of the receiver.

All three of the variables are known: the time and the frequency are those measured, while the amplitude is measured at the output as *M*(*f*,*t*,*a*). It should also be noted that the ratio *M*/*F_rx_* gives the input power to the receiver (for a given set of variables *t*, *f*, and *a*).

**Figure 5:**
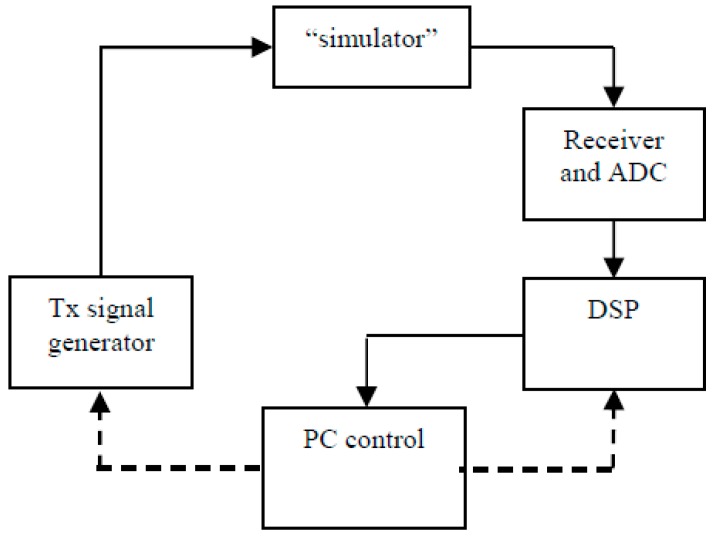
The setup used for calibrating only the receiver, to measure its input-output transfer function. A perfectly known signal must be inserted to the receiver input. It is obtained directly from the Tx signal generator (without the power amplifier) through an external attenuator, which acts as a “simulator” of the ionosphere attenuation.

To calibrate the system the terms *P_t_*, *A_ant_*, and *A_cable_* must be characterised: *P_t_* and *A_cable_* can be measured, *A_ant_* can be determined by means of simulations; the measurement of *F_rx_* is more difficult because of the dependence on amplitude, so a specific setup was conceived; it is displayed in Figure 5. The block “simulator” is simply a variable attenuator which can “simulate” the attenuation caused by the ionosphere. The aim is to input a set of signals in the receiver that are perfectly known and then observe the output. This is achieved by establishing a precise characterisation of the Tx signal generator output. The attenuator is a very precise instrument with a much higher accuracy than required for the measurements. It is important to note that a normal signal generator was not used to characterise the receiver because the signal generator inside the ionosonde provides the actual signal that is used for soundings and reproducing this exact signal would have been difficult or impossible with an external instrument.

Given the high stability of the various sections of the system, the only source of uncertainty is the receiver, but during calibration it was verified that the accuracy is well beyond that required for good measurement of ionospheric attenuation. If the relative measurements were carried out, the dependence on some of the terms appearing in (1) would disappear. Using the subscripts “1” and “2” to denote two different measurements taken in different times at the same frequency *f*, gives:

(2) $$\frac{M_{1}(f,t_{1},a_{1})}{M_{2}(f,t_{2},a_{2})}=\frac{P_{t}(f)\cdot A_{g}(f,t_{2})\cdot A_{ion}(f,t_{2})\cdot A_{ant}(f)\cdot A_{cable}(f)}{P_{t}(f)\cdot A_{g}(f,t_{1})\cdot A_{ion}(f,t_{1})\cdot A_{ant}(f)\cdot A_{cable}(f)}\cdot \frac{F_{rx}(f,a_{1})}{F_{rx}(f,a_{2})}\;\mathrm{and}:\;\frac{A_{g}(f,t_{2})\cdot A_{ion}(f,t_{2})}{A_{g}(f,t_{1})\cdot A_{ion}(f,t_{1})}=\frac{M_{1}(f,t_{1},a_{1})}{M_{2}(f,t_{2},a_{2})}\cdot \frac{F_{rx}(f,a_{2})}{F_{rx}(f,a_{1})}$$

Expression (2) simply shows that the ratio between the attenuations at two different times is equal to the ratio of the input powers at the receiver at the same times (*M*/*F_rx_*). Measuring *M*_1_ and *M*_2_ and using the receiver calibration function *F_rx_* it is possible to calculate the overall attenuation. If the geometric attenuation is known, then it is possible to calculate the intrinsic attenuation.

The difference between the situations described by Equations (1) and (2) is that the former allows for calculation of an absolute attenuation, and is affected by an imperfectly known systematic error due to antennas gain determination, the latter allows for calculation of relative attenuations and is not influenced by antenna characteristics, making it useful for monitoring variation in attenuation over specified time periods.

## 4. Calibration of the AIS-INGV Ionosonde

### 4.1. Receiver Calibration

The calibration of the receiver represents the longest part of the overall calibration process. The aim is to acquire the input-output characteristic, named *F_rx_*(*f*,*a*) in Equation (2), by means of the setup described in Figure 5.

Before starting calibration, the operative working parameters of the receiver had to be chosen. An internal attenuation and a value for the integrations have to be selected. Usually, the AIS works well with an internal attenuation of 2 dB and 30 integrations, so these were the values chosen for calibration. Calibration carried out with the cited parameter values is a valid starting point, allowing power measurements via the AIS ionosonde in the most likely conditions.

*F_rx_* depends on both frequency and input signal amplitudes. This means that it is necessary to make measurements not only at the various frequencies used during the sounding process, but also to simulate the various expected input amplitudes. Usually a receiver should exhibit a linear behaviour and, if this was the case, it would be sufficient to make measurements for only a couple of input levels, the minimum to establish the linear response parameters. In reality, the receiver exhibits a linear behaviour only in the middle of the range of operating conditions. Even though it is not frequent to receive very strong or very weak signals, we wanted to detect the response of the receiver in all possible conditions, and at the same time avoid any errors due to any possible deviations from linear behaviour (also in the segment thought to be linear).

For this reason, the “simulator” in Figure 5 has been arranged to stimulate the receiver over a wide range of input powers, but a limited number of input powers had to be selected. Using the external attenuator, it was decided to select a 2 dB “step” between input powers. This was a compromise value: it is small enough to make the calibration accurate but not so small that it would make the calibration procedure last too long. For all values not exactly the same as the ones measured during calibration, a linear interpolation is made when the power measurement is performed. As regards sounding frequencies the situation is different because sounding is performed at specified frequency values, say 1–20 MHz, with steps of 50 kHz. So, it is possible to repeat the calibration for all the exact frequency values that are to be used during sounding.

In order to establish an input-output relationship, it is necessary to know the input and output values. The outputs are simply read on the PC while the acquisition program runs. The usual sounding software used to produce ionograms (as in Figure 2) is not used, with instead a specific program that shows the amplitude at the receiver output for individual frequencies (very similar to what is shown in Figure 3).

**Figure 6:**
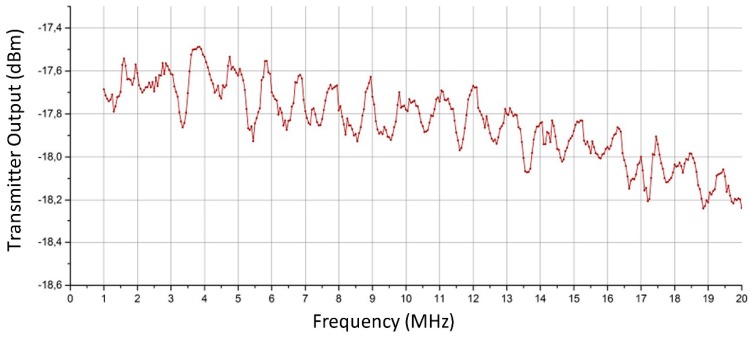
Signal power characterisation at the transmitter output.

The input powers had to be calculated starting from a knowledge of the Modulated Radio Frequency signal level (see Figure 1, considering that during calibration the power amplifier is not used) and subtracting the values for external attenuation. Figure 6 shows the output of the transmitter versus frequency. Considering the signal power at the transmitter output and the 2 dB step chosen for the input signals, a range from 32 to 66 dB was set.

The combination of different frequencies and amplitudes for which calibration is to be performed produces about 7000 different measurements. In fact there are 381 different frequencies (from 1 to 20 MHz, 50 kHz step) and 18 different power steps (from 32 to 66 dB of external attenuation, 2 dB step). Table 1 sums up the calibration parameters introduced so far.

Since all the measurements are subject to random errors, each one was repeated an adequate number of times (100) in order to obtain significant statistics. A specific acquisition software was developed as a doctorate degree thesis [24], making it possible to repeat the 100 measurements and, contemporary, change frequency automatically. The changing of the external attenuation was left manual, switching a mechanical device.

**Figure 7:**
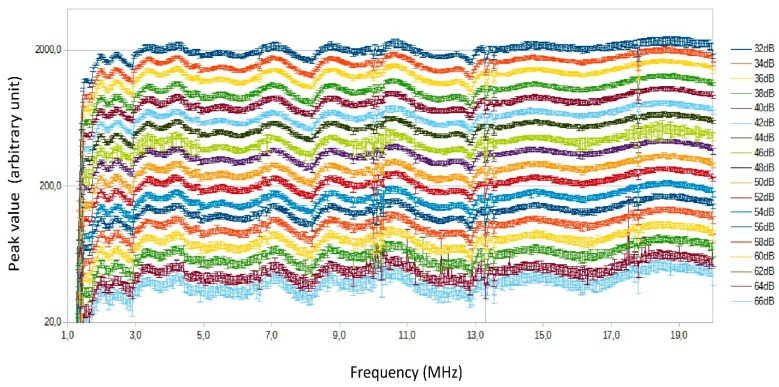
Receiver calibration curves. Each curve is the receiver response corresponding to a different value of external attenuation of the “simulator” in Figure 5. The vertical bars indicate the standard deviation.

A graphic representation of the set of acquired values is summarised in Figure 7. It is the representation of the conventional output values read on the acquisition program vs. frequency; each curve is associated to different external attenuation parameter and is identified by the different colours. The curves do not overlap, this is an evidence that the receiver has a monotonic response. It is possible to note also that, even considering the standard deviation, the curves do not overlap because of the proper choice of the attenuation step.

At some specific frequencies (e.g., 3, 10, 13.5 MHz) there are some anomalous rapid changes in response. These are due to minor imperfections in the receiver electronics but are without great significance because, in spite of their presence, the curves remain distinct. Also the fact that the curves indicate a poor response at frequencies lower than 1.5 MHz was expected and has not a great importance.

### 4.2. Overall System Calibration

In order to have all the relevant information to perform attenuation measurements, the calibration of the receiver has to be combined with a knowledge of the response of the other parts of the sounding system, namely (see Figure 4): the cables, the antennas, and the Tx power amplifier.

**Figure 8:**
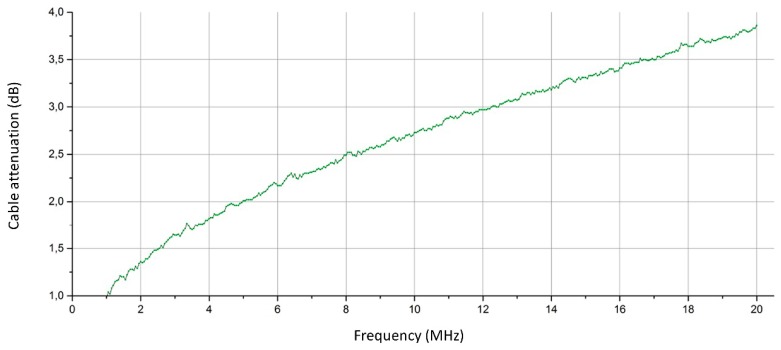
Characterisation of cable attenuation.

The attenuation of the cables (*A_cables_* in (1) and (2)) had to be taken into account because the sounding system is located quite far from the antenna system and cables of several tens of meters introduce a relevant degree of attenuation. This attenuation was measured by means of a network analyser and the result is displayed in Figure 8. There is only one trace because the attenuation of the two cables (in transmission and reception) were added together. It is seen that attenuation increases with frequency (as expected) and the absolute values are relatively small although not irrelevant.

**Figure 9:**
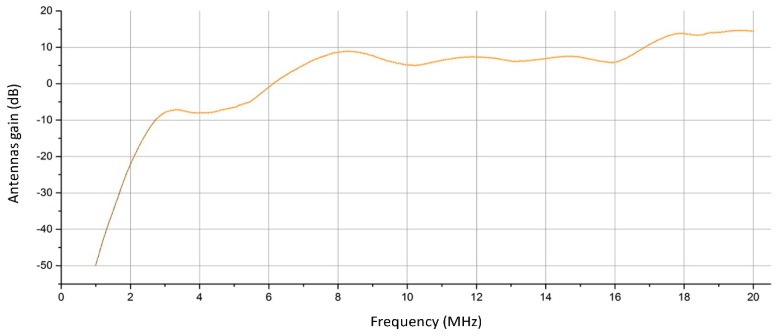
Antenna gain. The mismatch losses are very small and have been summed to the antenna gain to get a single curve. The negative values at some frequencies are normal for the specific antenna system used.

It was possible to measure the antenna impedance, which can be used to predict power loss due to impedance mismatches between the antennas and the cables. The contribution of the antennas (their gain) to the overall response was calculated by means of a simulation tool. The result of the sum of the gains of the two antennas and the mismatches is represented in Figure 9.

The gains in Figure 9 also take into account the calculated mismatches, based on the antenna impedance calculations and theoretical considerations. Note that the values are positive for frequencies greater than 6 MHz, otherwise they are negative, i.e., they are losses. This is a consequence of the particular type of antenna used for the ionosonde system: the delta antenna is a wide band progressive wave antenna providing an almost flat gain and an almost flat impedance over a very large bandwidth, with the disadvantage of poor response to the lower frequencies.

**Figure 10:**
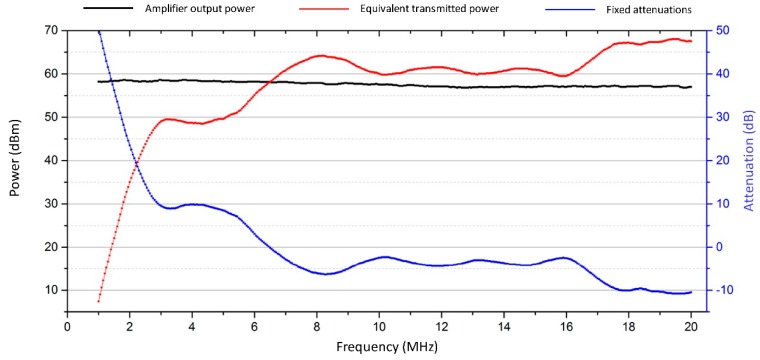
Transmitted equivalent power (in red), calculated subtracting all the fixed attenuations (in blue) from the amplifier output power (black line). The fixed attenuations are taken into account as the sum of the cable attenuation plus the antenna gain (here negative when the antenna gain is positive).

Concerning the power amplifier, it was simple to record the output characteristics of the one used by the AIS ionosonde using a spectrum analyser (the *P_t_* in Equations (1) and (2)). The result is shown in Figure 10, which includes all the preceding measurements.

The left vertical axis in Figure 10 shows power levels (in dBm), whilst the right axis shows attenuations (in dB). The black line represents the power amplifier output, which is almost constant across the entire band. The blue line represents all the “attenuations” present between the transmitter and the receiver (excluding the transmission medium, of course) obtained by subtracting the antenna gains from the cable attenuations. Note how this figure is negative for frequencies greater that approx. 6 MHz due to the antenna gains (negative attenuation is equivalent to a gain). The red trace is simply the difference between the black and the blue traces and represents a sort of a transmitted “equivalent” power. It embraces all contributions external to the receiver and medium attenuations, making them “collapse” into the transmitter. In this way, when an ionospheric attenuation measurement is to be performed, it is simply given by (in dB):

(3) $${\left[A_{ion+g}(f,t)\right]}_{dB}={\left[P_{eq}(f)\right]}_{dBm}-{\left[\frac{M(f,t,a)}{F_{rx}(f,a)}\right]}_{dBm}$$

Equation (3) is only a simplified version of Equation (1): *A_ion+g_* is the ionospheric attenuation under investigation (intrinsic plus geometric); *P_eq_* is the equivalent transmitted power defined above, and the ratio *M*/*F* is the power at the receiver input, as calculated by means of receiver calibration.

The corresponding form of Equation (2) for relative measurements (the difference between two measurements taken at times *t*_1_ and *t*_2_) is:

(4) $${\left[A_{ion+g}(f,t_{2})\right]}_{dB}-{\left[A_{ion+g}(f,t_{1})\right]}_{dB}={\left[\frac{M(f,t_{1},a)}{F_{rx}(f,a)}\right]}_{dB}-{\left[\frac{M(f,t_{2},a)}{F_{rx}(f,a)}\right]}_{dB}$$

## 5. Attenuation Measurements with the AIS-INGV Ionosonde

### 5.1. Overview

The aim of the authors is to make data available on the attenuation of signals travelling through the ionosphere in order to extract relevant information useful for advanced studies on the ionosphere. Once the AIS ionosonde has been properly calibrated, various methods of presentation of the measured attenuation values can be conceived. Schematically, there are two main types of sounding:

- ionograms, i.e., a scan over a range of frequencies to extract the virtual heights of the reflecting layers;
- single frequency soundings, performed to monitor some particular predicted phenomenon over a period of time.

A knowledge of the strength of the received signal can be inserted into each of the preceding procedures and, for the second case, it is possible to choose between two different forms of representation. In the following paragraphs some examples of the mentioned types of measurement will be shown, taking the measures in some typical conditions.

### 5.2. Enriched Ionogram

Information on ionospheric absorption can be included in ionograms using different colours for different signal levels. To obtain this output, the sounding software has to be modified according to the following logical scheme:

- for each frequency, apply the calibration coefficient to determine the overall attenuation of the received signal (performing linear interpolation for values not included among those used during calibration);
- make an average on many measurements;
- take into account (possible) geometric attenuation, based on the virtual height of reflection (this type of calculation has not yet been performed);
- mark a point on the ionogram corresponding to the sounded frequency/height pair; each point has a colour, chosen from a colour scale, depending upon the calculated attenuation;
- repeat the previous steps using different frequencies, until the whole range of frequencies has been scanned; while scanning all displayed data are also stored in a file.

**Figure 11:**
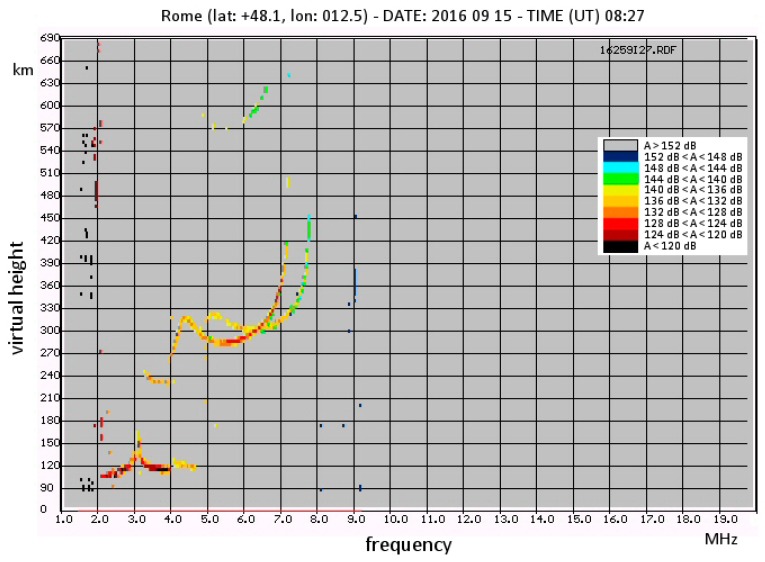
Example of an enriched ionogram. With respect to a conventional ionogram each point of the traces has a colour associated to a different value of the overall attenuation (here it includes the geometric attenuation) using the calibration coefficients.

The implementation of software for this type of output was conducted during a doctorate degree thesis [24]; an example ionogram “enriched” as described is displayed in Figure 11.

All the usual traces are present, but not in black and white (as in Figure 2). The colour scale is drawn on the upper-right end of the ionogram. It can be seen that attenuation is less for the lower layers (E layer) and becomes increasingly higher as the frequency approaches the critical F2 frequency.

Note that the ionogram is a representation of the height of reflection versus frequency (with attenuation as a parameter) performed at a fixed time (the time the ionosonde takes to perform the sounding is negligible relative to the typical variation times of the ionosphere). The computed attenuations are absolute (no relative evaluation is possible because an ionogram “freezes” the situation at a certain moment). The main interest of this output is to provide a quick estimate of overall attenuation: the colour scale step is much greater than the possible systematic error due to imperfect knowledge of the antenna responses.

### 5.3. Single Frequency Study of a Specified Layer

If the focus of interest is the evolution of the characteristics of a specific layer, then an ionogram is not the best sounding output. In this case a single frequency sounding is preferable, using a signal frequency known to be reflected by that layer. This type of sounding outputs a graph with time on the x-axis rather than frequency, and attenuation on the y-axis. The height of reflection is assumed to be fixed, although this obviously might not always be true and so “gating” has to be implemented to exclude possible echoes from different heights. The logical scheme to be executed by the software is as follows:

- once a frequency has been chosen, acquire numerous echoes, recording the delay (height) and amplitude;
- exclude all echoes not coming from a specified (narrow) interval of heights;
- apply the calibration data to calculate attenuation;
- take geometric attenuation into account for the specified height (the interval of heights is assumed small enough so that geometric attenuation can be considered constant across it);
- mark a point on the output graph corresponding to the sounding time and calculated attenuation;
- wait a specified interval of time and repeat the measurement.

**Figure 12:**
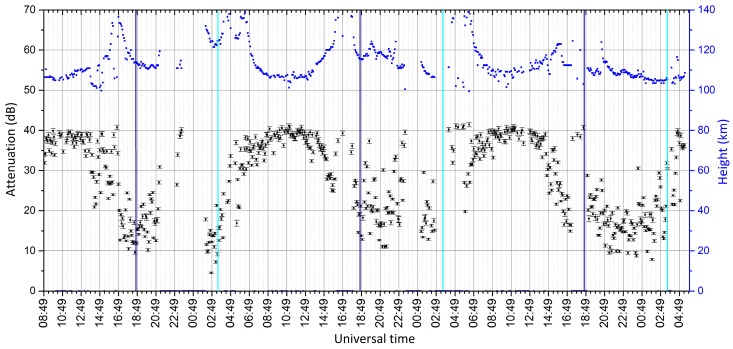
Example of a single frequency-single layer study over a three days period. The blue dots represent the heights of the selected echoes (vertical axis on the right); the black dots represent the ionospheric attenuation, computed after subtracting the geometrical attenuation (vertical axis on the left).

An example of this type of graphic output is shown in Figure 12.

The figure includes two graphs: the black dots represent the main information about attenuations (vertical axis on the left), the blue dots represent the exact height of each echo (vertical axis on the right) measured at the same times (x-axis in UT, Universal Time = Local Time − 1). A dispersion bar is added for each black dot, deduced from the calibration uncertainties (see Figure 7). This sounding was performed starting from 2 September 2016 at 8.49 (UT), until 4 September 2016 at 5.49 (UT), repeating sounding every 5 min, using a frequency of 2.3 MHz and a selected height interval of 95–140 km.

The cyan/blue vertical lines represent the moments of the sunrise/sunset, estimated for a height of 75 km (this is the reason why the daytime is longer than night despite the date is close to the equinox). The reason sunrise and sunset were estimated at 75 km is because the main contribution to attenuation is thought to be absorption by the D layer, while the contribution of the E layer (where reflection occurs) is expected to be negligible. It can be seen that attenuation during the day increases with the height of the sun above the horizon, while during the night the D and E layers disappear or some echo is present from a sporadic-E layer, which is much less reliable and, when present, causes low attenuation.

This type of graph is useful for monitoring the temporal trend for a particular layer. The attenuations are absolute but, even though their values are affected by errors, the differences are not, so the evaluation of the attenuation trend is reliable.

### 5.4. Single Frequency Study of the Whole Ionosphere

**Figure 13:**
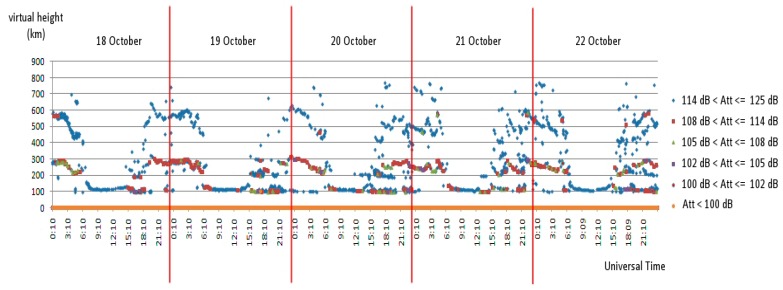
Example of a single frequency-all layer study over a five days period. With respect to Figure 12 all layers are visible at a glance, while the attenuation is represented by different colours (now the overall attenuation is considered, including the geometrical one).

It is possible to monitor echoes from all heights, but, unlike the previous case, the output must indicate the height value for each echo. The result is a graph with the heights on the vertical axis, like an ionogram, and the measurement time on the x-axis. As in the enriched ionogram, attenuation is represented by a colour scale. The difference from an ionogram is that only a single frequency is sounded, with a single graph depicting the evolution of the entire ionosphere, so while an ionogram freezes all frequencies at a single moment, this output freezes all times at a single frequency. An example of this type of graph is shown in Figure 13 (in this case displaying global attenuation, including geometrical attenuation).

### 5.5. A Possible Further Elaboration of Single Layer Measurements

Even though this paper has the purpose of presenting a data acquisition technique regarding ionospheric attenuation, without particular scientific aims, a simple elaboration of the raw data was carried out as an illustrative example of the possibilities for this technique.

**Figure 14:**
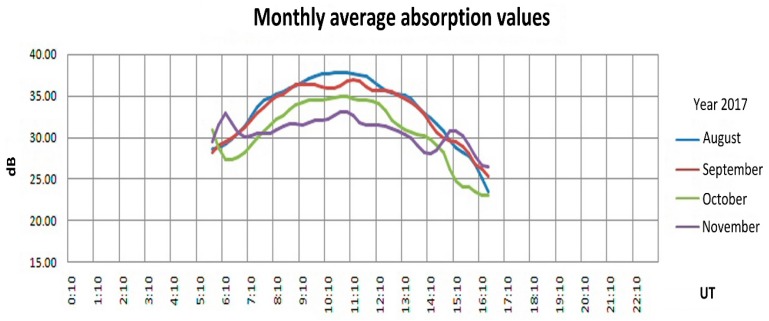
Example of monthly median absorption at 2.3 MHz. Each line represents the attenuations as in Figure 12, averaging the values along a month (only echoes from 95 to 140 km are considered).

The raw attenuation data for a single frequency was acquired over a number of months, of the same type as shown in Figure 12. The frequency used was 2.3 MHz and attention was focused only on daytime hours when the D and E layers are more stable (at night the Es layer can be present on an irregular basis). The attenuation values measured at the various hours were averaged (median value) for all the days of each month, from August to November 2017. The result is displayed in Figure 14, in which each curve refers to a different month, on the horizontal axis there is the hour (UT) and on the vertical axis the ionospheric attenuation (after subtracting geometric attenuation).

The curves extend only from approx. 5:30 to 16:30 UT because of the cited interest only in daytime hours. Even after such a simple study it is possible to note that in all the months the maximum attenuation occurs at noon (local time), mostly due to the D layer. Moreover, passing from August to the autumn months the insolation decreases, as does the D layer’s absorption. Curves like those in Figure 14 can be taken as representative of a “quiet” ionosphere, and used as a reference to highlight deviations under particular conditions (i.e., storms or other anomalies).

## 6. Notes on Measurement Accuracy

The measurements carried out by the AIS ionosonde, like all measurements, are affected by errors that now will be evaluated. There are two general types of causes for errors:

- random fluctuations in the measurements registered by the receiver;
- uncertainties about the system characteristic parameters, which are almost constant at least over many weeks or months.

All errors are expressed in dB, which is the most suitable unit when dealing with gains or attenuations and so it is convenient also to express errors in the same unit. Given that dBs express ratios, it is possible to demonstrate that the error figures, known and expressed in dB, can be combined in the same way as if they were relative errors (in linear units), for which the resulting error is given as a quadratic average. In other words, to combine two errors, characterised by a standard deviation in dB equal to *E_dB_*_1_ e *E_dB_*_2_, the resulting error can easily be calculated with:

(5) $$E_{dBtot}=\sqrt{E_{dB1}{}^{2}+E_{dB2}{}^{2}}$$

The first type of error can be minimised by calculating averages (a simple average of linear values). During calibration output fluctuations were observed (see Figure 7) and it is possible to establish a standard deviation for this type of error of about 0.7 dB (or less).

Analysing the second type of errors is more difficult, because they can be included among the systematic errors. It is assumed that the measured system parameter values (transmitted power, cable attenuation, etc.) are not precisely known but remain constant. To increase the measurement reliability, the calibration and measurements are carried out in the most controlled and stable conditions possible, for example waiting for the thermal stabilisation of the equipment. Analysing this type of error in more detail, three error categories can be defined:

(a) deriving from direct measurement,
(b) deriving from indirect measurement,
(c) not determined by measurement, but indirectly evaluated by means of computer simulations.

The errors in category (a) are due to the accuracy of the laboratory instrumentation; this item of data is considered to be 0.05 dB. There are two different measurements: cable attenuations and transmitted power (the Tx and Rx cables were established in a single measurement, connecting them in cascade), so the resulting error is given by (5): 0.05 √2 = 0.07 dB, quite small, so that it could be ignored relative to all other errors.

Category (b) includes measurements of attenuations due to cable-antenna mismatches. These mismatches cause a small attenuation of the energy flowing from the transmitter to the transmitting antenna, and from the receiving antenna to the receiver. It is difficult to directly measure this mismatch because of the particular antenna configuration, so mismatch attenuation is determined by means of calculations based on the measurement of the reflected energy at the cable-antenna transition, measured at the other end of the cable. Ultimately the error is evaluated by suitable analysis of error propagation using the conversion formula. This analysis produced a very small value, about 0.01 dB, so it can also be ignored.

Type (c) errors are the most complicated to evaluate, since it is impossible a directly measure the antenna gain, so this has to be determined using computer simulations. The antenna’s characteristics are well known (cable positions, dimensions, and materials) and if they were located in open space or above an ideal ground (perfectly conductive) the evaluations would be very precise. Unfortunately there are many factors that cause antennas to deviate from a free space behaviour, the main ones being the characteristics of the terrain and the presence of obstacles. Some structures (buildings, trees, other antennas, and the antenna masts used to hold them in position) near antennas can alter their radiation pattern and simulating these effects is somewhat difficult. The behaviour of the terrain is easier to deal with, but it varies over time, mainly due to changes in humidity.

The assessment of antenna gain performed during the calibration procedure on the AIS ionosonde was carried out without taking into account nearby structures but only the ground properties. It was important to evaluate, at least roughly, the effects of variations in the terrain, so the consequences of ground property variations were evaluated within wide ranges (starting from mean values, the electric permittivity was varied by ±20% and conductivity by ±50%). The results gave an estimation of the error in antenna gains (the sum of the gains of the two antennas) within 0.8 dB (but this varies with frequency). This type of error cannot be properly defined as systematic, because ground properties can vary within a few hours.

Even though the nearby structures were not taken into account, at least an approximate evaluation of their influence needed to be made, so the presence of an adjacent antenna positioned near the active one was assessed (the second antenna is used in different times, so it is considered passive when AIS is active). It is thought that the effect of the second antenna is probably the most significant one compared to the other structures, and it influences the overall gain by 2.5 dB.

Table 2 summarises all the figures mentioned in this section.

The estimation of the overall error for the absolute measurements is thus:

$$E_{dBtot}=\sqrt{0.7^{2}+0.07^{2}+0.8^{2}+2.5^{2}}=\pm 2.7\;\mathrm{dB}\;\;(absolute\;error).$$

Finally, it needs to be recalled that all systematic errors should only be considered for the absolute measurements, while for the relative measurements (Equations (2) and (4)) they cancel out and only the random receiver errors remain (considered twice), plus those due to the terrain. So the residual error is:

$$E_{dBtot}=\sqrt{0.7^{2}+0.7^{2}+0.8^{2}}=\pm 1.3\;\mathrm{dB}\;\;(relative\;error)$$

These conclusions are interesting because they show that the main component of the absolute error is due to the imperfect characterisation of the antennas. If in the future simulations for estimating antenna gain can be refined, this error could be reduced to about 1 dB.

This study, even if approximate, is useful because it establishes a lower limit to the attenuations values that can be studied with the AIS system. In fact, even the current 2–3 dB errors are acceptable considering the 2 dB calibration quantum and the ionospheric attenuation variations being measured, which can vary across wide ranges, even up to 30 dB.

## 7. Conclusions

The availability of the measure of the ionospheric attenuation can make it possible to better study some phenomena occurring in the ionosphere. Only to mention some of them: the characteristics of the D layer will be inspected better, given that now they can be inferred only by means of models based on other measurements; also the E-sporadic layer can be studied in more detail; having an instrument that performs continuous measures, possible occurrence of solar storms could be followed with an additional method of tracing.

In this work a method is presented for measuring ionospheric absorption by means of direct measurement of reflected energy in a vertical sounding. The measurement is accomplished by comparing transmitted and received energy using the AIS ionosonde at the Rome ionospheric observatory. The ratio of the two energies must take into account the characteristics of the various elements comprising the AIS system: receiver response, cable attenuations, and antenna gains. Two types of measures are possible, absolute and relative.

This direct measurement represents an advantage with respect to other methods of measuring ionospheric attenuation because it does not involve multiple reflections, which would require knowledge of the reflection coefficient of the terrain.

The evaluation of the predicted error for absolute measurement of ionospheric absorption produced a value of ±2.7 dB, mostly due to systematic errors introduced by the imperfect knowledge of the antenna gains. Performing a relative measurement (i.e., comparing absorption in different times) can cancel out the systematic errors, lowering the predicted error to ±1.3 dB.

Among future developments can be cited: a better characterisation of antennas, performing calibration using different operative parameters for the ionosonde, an automation of the calibration procedure. It was seen that the not perfect knowledge of the antenna gains is the main source of uncertainty in the measurement; a better modelling of the antenna characteristics could reduce the absolute error to a value of around 1 dB that would be very interesting for studying the properties of the quiet ionosphere and monitoring possible anomalies. Repeating the calibration for different operative parameters of the AIS ionosonde would be useful for a good performance in different operative conditions (the site or special measurement campaigns). In these cases the automation of the calibration procedure would result particularly useful.

## Figures and Tables

**Table 1:** Summary of the parameters values used for the calibration. The frequency range and step are the usual ones used in standard vertical sounding. The internal attenuation and integration factor were established and maintained constant after empirical experience. The external attenuation range was chosen considering the transmitted output power, while the step value was selected to get a limited number of different measurements.

Parameter	Value (s)	Units
Frequency range	1 to 20	MHz
Frequency step	50	kHz
Internal attenuation	2	dB
Integration factor	30	(number)
External attenuation	32 to 66	dB
External atten. step	2	dB

**Table 2:** Standard deviations of the relevant measurements.

Measurement/Evaluation	s.d. (dB)	Note
receiver random error	0.7	
direct measurement	0.07	ignored
indirect measurement	0.01	ignored
ground variations	0.8	
nearby structures	2.5	
